# Comparative analysis of *POD* genes and their expression under multiple hormones in *Pyrus bretschenedri*

**DOI:** 10.1186/s12863-024-01229-7

**Published:** 2024-05-06

**Authors:** Guohui Li, Muhammad Aamir Manzoor, Guoyu Wang, Shiping Huang, Xiaoyuan Ding, Muhammad Abdullah, Ming Zhang, Cheng Song

**Affiliations:** 1 Anhui Provincial Key Laboratory for Quality Evaluation and Improvement of Traditional Chinese Medicine, College of Biological and Pharmaceutical Engineering, West Anhui University, Lu’an, 237012 China; 2 Department of Plant Science, School of Agriculture and Biology, Shanghai Jiao Tong University, Shanghai, China; 3 College of Pharmacy, Anhui University of Chinese Medicine, Hefei, China; 4 Queensland Alliance of Agriculture and Food Innovation, The University of Queensland, Brisbane, 4072 Australia

**Keywords:** Class III peroxidase (POD), Genome-wide analysis, *Pyrus bretschenedri*, Abiotic stress, Expression analysis

## Abstract

**Background:**

Class III peroxidase (POD) enzymes play vital roles in plant development, hormone signaling, and stress responses. Despite extensive research on POD families in various plant species, the knowledge regarding the POD family in Chinese pear (*Pyrus bretschenedri*) is notably limited.

**Results:**

We systematically characterized 113 POD family genes, designated as *PbPOD1* to *PbPOD113* based on their chromosomal locations. Phylogenetic analysis categorized these genes into seven distinct subfamilies (I to VII). The segmental duplication events were identified as a prevalent mechanism driving the expansion of the *POD* gene family. Microsynteny analysis, involving comparisons with *Pyrus bretschenedri*, *Fragaria vesca*, *Prunus avium*, *Prunus mume* and *Prunus persica*, highlighted the conservation of duplicated POD regions and their persistence through purifying selection during the evolutionary process. The expression patterns of *PbPOD* genes were performed across various plant organs and diverse fruit development stages using transcriptomic data. Furthermore, we identified stress-related *cis*-acting elements within the promoters of *PbPOD* genes, underscoring their involvement in hormonal and environmental stress responses. Notably, qRT-PCR analyses revealed distinctive expression patterns of *PbPOD* genes in response to melatonin (MEL), salicylic acid (SA), abscisic acid (ABA), and methyl jasmonate (MeJA), reflecting their responsiveness to abiotic stress and their role in fruit growth and development.

**Conclusions:**

In this study, we investigated the potential functions and evolutionary dynamics of *PbPOD* genes in *Pyrus bretschenedri*, positioning them as promising candidates for further research and valuable indicators for enhancing fruit quality through molecular breeding strategies.

**Supplementary Information:**

The online version contains supplementary material available at 10.1186/s12863-024-01229-7.

## Background

Peroxidases (PODs) occur in a wide range of living organisms and are considered a diverse multigene family [1, 2]. With the use of hydrogen peroxides as an electron acceptor in their active center with a metal, peroxidases are known to catalyze oxidative reactions [3]. Heme PODs, along with nonheme PODs are two major groups of peroxidases based on variations in their structure. Two subfamilies (e.g., animal PODs, alongside nonanimal PODs) comprise the heme PODs whereas three major classes viz., class I, class II, and class III, comprise the nonanimal superfamily [1, 4]. Class III peroxidases act as plant-specific oxidoreductases and various studies have abbreviated the class III peroxidases in various ways, such as PER, Px, POX, Prx, and POD [5]. POD is the abbreviation that is used in this study, and it is basically a plant-specific oxidoreductase. PODs are widely distributed in microorganisms, plants, and animals [2]. In terms of plant growth, PODs are known for their dual function, as they can both harden and soften plant cell walls, and have been reported to play roles various processes, such as germination, lignification, development as well and plant defense via various action mechanisms, for example the formation of radicals, the regulation of ROS, and substrate oxidation [3].

With the advent of transcriptomic analysis, large numbers of PODs, which are known to perform different functions, have been identified. The role of PODs is still elusive, with only a few studies present in the literature highlight the functional role of PODs [7]. Moreover, cold stress resistance improved with the *POD* (*AtPrx69*, *AtPrx22*, and *AtPrx39*) gene overexpression in *Arabidopsis thaliana* [7]. However, the *POD* gene in cotton, namely GhPOX1 is known for its ability to increase ROS production [8]. The regulation of the *POD* genes in *Zea mays* (roots) are regulated by salicylic acid, methyl jasmonate, and pathogen elicitors [9]. According to various studies, *POD* genes are primarily involved in resisting or responding to stress stimuli in addition to playing some physiological and biological roles [10].

Bioinformatics analysis has been majorly used to study and characterize the number of POD in various, plants including 73 PODs in *Arabidopsis thaliana*; 93 in *Populus trichocarpa*, 138 in one of the important serial crop species, e.g., *Oryza sativa*; 119 in *Zea mays*; and 102 in *Medicago sativa* [3, 11]. The demand for pear (*Pyrus* species) fruit demand has increased around the world due to its low price and health benefits [12]. A wide range of bioinformatics analysis were performed in this study for the *POD* gene family; moreover, these genes play pivotal roles in helping plants respond to or resist various stress stimuli. In total, 113 genes were identified for the first time in the pear genome, and this analysis was performed with the aid of genome-wide approaches.

In this study, we have considered chromosomal mapping, physicochemical properties, gene duplication events, phylogenetic relationships, collinear correlation, rate of substitution, GO and KEGG enrichment analysis, promoter sequence analysis, and expression profiling in response to various conditions under melatonin (MEL), salicylic acid (SA), abscisic acid (ABA), and methyl jasmonate (MeJA) stress. The present study contributes to the future enhancement of crop and fruit quality, providing a deeper understanding of the various *POD* genes. Research on pears is of utmost importance because it lays the foundation for improving the cultivation of this fruit. Therefore, the results obtained in this study may lead to advancements in the characterization of this species/genus, ultimately benefiting fruit quality. Our research is designed to offer a comprehensive global classification and analysis of plant gene families.

## Results

POD gene family identification and characterization in *P. bretschneideri.* In this study, we identified a total of 113 *POD* genes within the *P. bretschneideri* genome, for simplicity, we designated them as *PbPOD1* through *PbPOD113* based on their corresponding chromosomal position. Additionally, we delved into valuable details about these PODs, including their protein identifiers, where they are located on the chromosomes, the length of their coding sequences (CDS) in base pairs, and various physical attributes, such as isoelectric points (pIs), molecular weight in kilodaltons (kDa), as well as protein length in amino acids (aa), and the grand average of hydropathicity (GRAVY).

While protein length varied between 83 amino acids (PbPOD36) and 1314 amino acids (PbPOD66) with an average of 335.22 amino acids. Similarly, Molecular weight ranged between 9017.36 kDa (PbPOD36) and 143415.68 kDa (PbPOD66) with a mean value of 36.61 kDa. On the other side, isoelectric points ranged between 4.29 (PbPOD11) and 9.73 (PbPOD35). The GRAVY results displayed diversity, with values spanning from − 1.001 (PbPOD30) to 0.124 (PbPOD14). It is noteworthy that most of these genes exhibited hydrophilic properties, with 15 genes demonstrating hydrophobic characteristics by displaying positive GRAVY values (Table 1).

**Table 1:** Characterization of *POD* genes in *P. bretschneideri*

Gene name	Gene ID	Number of amino acids	MW (kDa)	pI	Aliphatic index	GRAVY
***PbPOD1***	**Pbr032785.1**	301	32.91	8.13	83.95	-0.104
***PbPOD2***	**Pbr035186.1**	325	35.27	8.82	84	-0.092
***PbPOD3***	**Pbr040489.1**	331	36.41	9.33	84.59	-0.224
***PbPOD4***	**Pbr023311.1**	284	31.612	9.04	86.55	-0.349
***PbPOD5***	**Pbr003171.1**	103	11.18	7.85	82.52	-0.212
***PbPOD6***	**Pbr022808.1**	314	34.17	5.61	76.82	-0.078
***PbPOD7***	**Pbr022809.1**	95	10.49	7.78	107.79	0.037
***PbPOD8***	**Pbr021747.1**	354	Undefined	Undefined	83.76	-0.012
***PbPOD9***	**Pbr000686.1**	332	35.77	8.36	90.84	0.075
***PbPOD10***	**Pbr000687.1**	324	34.72	4.48	79.48	-0.207
***PbPOD11***	**Pbr000689.1**	350	37.45	4.29	87.29	-0.009
***PbPOD12***	**Pbr000691.1**	350	37.09	4.48	85.06	0.026
***PbPOD13***	**Pbr013214.1**	341	37.35	4.81	93.75	0.048
***PbPOD14***	**Pbr013078.1**	319	34.39	6.23	90.22	0.124
***PbPOD15***	**Pbr013077.1**	330	35.89	6.59	84.58	-0.205
***PbPOD16***	**Pbr013075.1**	327	35.04	9.29	80.55	-0.181
***PbPOD17***	**Pbr033934.1**	352	38.48	5.62	75.43	-0.394
***PbPOD18***	**Pbr003832.1**	332	35.67	6.88	79.1	-0.077
***PbPOD19***	**Pbr006566.1**	153	16.92	9.47	94.25	-0.073
***PbPOD20***	**Pbr032800.1**	341	38.51	8.93	80.88	-0.31
***PbPOD21***	**Pbr041097.1**	405	44.47	6.03	75.11	-0.303
***PbPOD22***	**Pbr002505.1**	352	39.18	5.91	85.88	-0.298
***PbPOD23***	**Pbr002542.1**	346	38.60	9.05	95.81	-0.089
***PbPOD24***	**Pbr000438.1**	391	42.97	9.22	81.61	-0.198
***PbPOD25***	**Pbr000146.1**	341	38.74	8.3	78.89	-0.436
***PbPOD26***	**Pbr014180.2**	215	23.39	5.11	70.84	-0.405
***PbPOD27***	**Pbr013845.1**	868	98.12	6.33	80.16	-0.539
***PbPOD28***	**Pbr010973.1**	318	34.61	9.43	89.31	-0.112
***PbPOD29***	**Pbr010975.1**	329	35.67	9.65	78.02	-0.265
***PbPOD30***	**Pbr010976.1**	142	16.18	8.44	53.59	-1.001
***PbPOD31***	**Pbr010977.1**	161	18.18	9.11	68.39	-0.572
***PbPOD32***	**Pbr002947.1**	109	12.01	5.34	75.96	-0.144
***PbPOD33***	**Pbr002948.1**	471	51.55	8.99	70.04	-0.404
***PbPOD34***	**Pbr002950.1**	318	34.58	9.31	89.31	-0.111
***PbPOD35***	**Pbr002956.1**	329	35.81	9.73	77.72	-0.259
***PbPOD36***	**Pbr002957.1**	83	90.17	6.54	80.96	-0.135
***PbPOD37***	**Pbr013905.1**	336	37.24	8.72	84.17	-0.121
***PbPOD38***	**Pbr040033.1**	328	35.55	9.02	80	-0.156
***PbPOD39***	**Pbr026505.1**	331	35.93	8.79	85.74	-0.073
***PbPOD40***	**Pbr026504.1**	331	36.04	8.89	83.96	-0.067
***PbPOD41***	**Pbr026503.1**	331	36.04	8.62	84.26	-0.051
***PbPOD42***	**Pbr026502.1**	327	35.58	8.57	86.18	-0.108
***PbPOD43***	**Pbr004299.1**	310	33.40	4.82	92.84	0.168
***PbPOD44***	**Pbr020588.1**	323	34.41	5.88	92.17	0.062
***PbPOD45***	**Pbr020590.1**	323	34.41	5.88	92.17	0.062
***PbPOD46***	**Pbr006117.1**	321	34.70	8.11	90.22	-0.002
***PbPOD47***	**Pbr006119.1**	321	34.72	8.41	90.22	-0.012
***PbPOD48***	**Pbr036549.1**	337	36.12	5.44	81.39	0.009
***PbPOD49***	**Pbr036474.1**	323	34.75	5.32	83.96	-0.036
***PbPOD50***	**Pbr005400.1**	318	34.37	8.71	81.73	-0.007
***PbPOD51***	**Pbr008699.1**	450	49.35	5.63	74.29	-0.259
***PbPOD52***	**Pbr018082.1**	327	35.48	9.08	82.91	-0.127
***PbPOD53***	**Pbr018080.1**	326	35.60	9.01	89.79	-0.097
***PbPOD54***	**Pbr026235.1**	327	35.94	8.58	80.61	-0.082
***PbPOD55***	**Pbr010213.1**	356	39.61	5.44	84.63	-0.284
***PbPOD56***	**Pbr010258.1**	498	56.14	8.2	89.28	-0.307
***PbPOD57***	**Pbr010270.1**	346	38.62	9.26	95.26	-0.105
***PbPOD58***	**Pbr031894.1**	336	38.34	8.3	80.95	-0.428
***PbPOD59***	**Pbr020734.1**	262	28.68	6.51	75.27	-0.494
***PbPOD60***	**Pbr020725.1**	262	28.68	6.51	75.27	-0.494
***PbPOD61***	**Pbr027164.1**	350	38.06	5.2	84.14	-0.195
***PbPOD62***	**Pbr022326.1**	301	32.77	7.54	72.36	-0.209
***PbPOD63***	**Pbr003310.1**	247	26.54	4.38	71.09	-0.45
***PbPOD64***	**Pbr003309.1**	324	34.78	4.57	77.93	-0.226
***PbPOD65***	**Pbr003308.1**	332	35.71	7.53	88.49	-0.032
***PbPOD66***	**Pbr011557.1**	1314	14.34	5.78	92.38	-0.245
***PbPOD67***	**Pbr011559.1**	320	34.90	8.97	90.25	-0.033
***PbPOD68***	**Pbr011560.1**	322	35.08	9.47	87.3	-0.212
***PbPOD69***	**Pbr011562.1**	314	33.54	9.35	78.57	-0.22
***PbPOD70***	**Pbr026058.1**	311	33.75	8.09	77.2	-0.261
***PbPOD71***	**Pbr014607.1**	308	32.69	8.91	85.29	-0.026
***PbPOD72***	**Pbr014605.1**	249	26.22	8.94	78.03	-0.226
***PbPOD73***	**Pbr008320.1**	156	17.36	5.82	76.35	-0.599
***PbPOD74***	**Pbr008291.1**	250	27.63	5.3	81.6	-0.398
***PbPOD75***	**Pbr035815.1**	338	35.70	5.1	82.57	-0.096
***PbPOD76***	**Pbr035513.1**	160	17.77	8.75	102.31	0.144
***PbPOD77***	**Pbr039193.1**	228	25.11	8.93	84.78	-0.167
***PbPOD78***	**Pbr030045.1**	338	36.94	4.81	96.6	0.038
***PbPOD79***	**Pbr015016.1**	333	35.74	4.42	88.77	-0.022
***PbPOD80***	**Pbr015032.1**	324	35.05	5.49	91.82	0.095
***PbPOD81***	**Pbr014793.1**	331	36.10	7.03	93.66	-0.049
***PbPOD82***	**Pbr034800.1**	318	34.33	9.37	82.23	-0.107
***PbPOD83***	**Pbr034821.1**	573	63.18	6.96	91.8	-0.239
***PbPOD84***	**Pbr016853.1**	277	29.54	6.41	81.7	-0.129
***PbPOD85***	**Pbr002672.1**	327	36.55	9.52	82.97	-0.242
***PbPOD86***	**Pbr005912.1**	864	93.87	7.87	92.95	-0.177
***PbPOD87***	**Pbr009308.1**	327	36.55	9.52	82.97	-0.242
***PbPOD88***	**Pbr027845.1**	330	35.36	5.4	77.52	-0.186
***PbPOD89***	**Pbr010632.1**	320	34.69	7.53	86.84	-0.096
***PbPOD90***	**Pbr007872.1**	339	37.26	5.36	78.82	-0.219
***PbPOD91***	**Pbr036152.1**	340	37.56	8.28	90.38	-0.144
***PbPOD92***	**Pbr036153.1**	336	36.76	8.03	89.7	-0.04
***PbPOD93***	**Pbr011189.1**	446	49.29	9.1	87.83	-0.15
***PbPOD94***	**Pbr006005.1**	311	33.75	8.09	77.2	-0.261
***PbPOD95***	**Pbr026772.1**	324	35.05	5.49	91.82	0.095
***PbPOD96***	**Pbr034488.2**	325	35.46	8.78	87.38	-0.124
***PbPOD97***	**Pbr034480.1**	326	35.43	9.01	88.31	-0.13
***PbPOD98***	**Pbr034479.1**	199	21.63	9.41	77.99	-0.293
***PbPOD99***	**Pbr041827.1**	326	35.73	8.82	77.24	-0.186
***PbPOD100***	**Pbr007903.1**	350	38.10	5.2	84.97	-0.181
***PbPOD101***	**Pbr007908.1**	325	35.82	6	82.34	-0.131
***PbPOD102***	**Pbr007909.1**	325	36.03	8.33	81.42	-0.14
***PbPOD103***	**Pbr037526.1**	329	36.38	5.91	85.35	-0.137
***PbPOD104***	**Pbr015968.1**	344	37.91	5.25	94.97	0.178
***PbPOD105***	**Pbr006343.1**	291	32.30	5.06	82.41	-0.153
***PbPOD106***	**Pbr037665.1**	295	31.95	7.53	83.36	-0.151
***PbPOD107***	**Pbr037664.1**	295	31.95	7.53	83.36	-0.151
***PbPOD108***	**Pbr015965.1**	291	31.04	7.55	84.16	-0.105
***PbPOD109***	**Pb015969.1**	109	11.82	5.1	82.2	0.205
***PbPOD110***	**Pbr027137.1**	414	45.08	7.71	78.31	-0.442
***PbPOD111***	**Pbr027136.1**	435	47.43	7.7	79.01	-0.441
***PbPOD112***	**Pbr000988.3**	255	27.80	5.82	74.71	-0.391
***PbPOD113***	**Pbr019188.1**	878	97.91	5.85	71.09	-0.647

### Phylogenetic relationship of *PbPOD* gene family

**Fig. 1:**
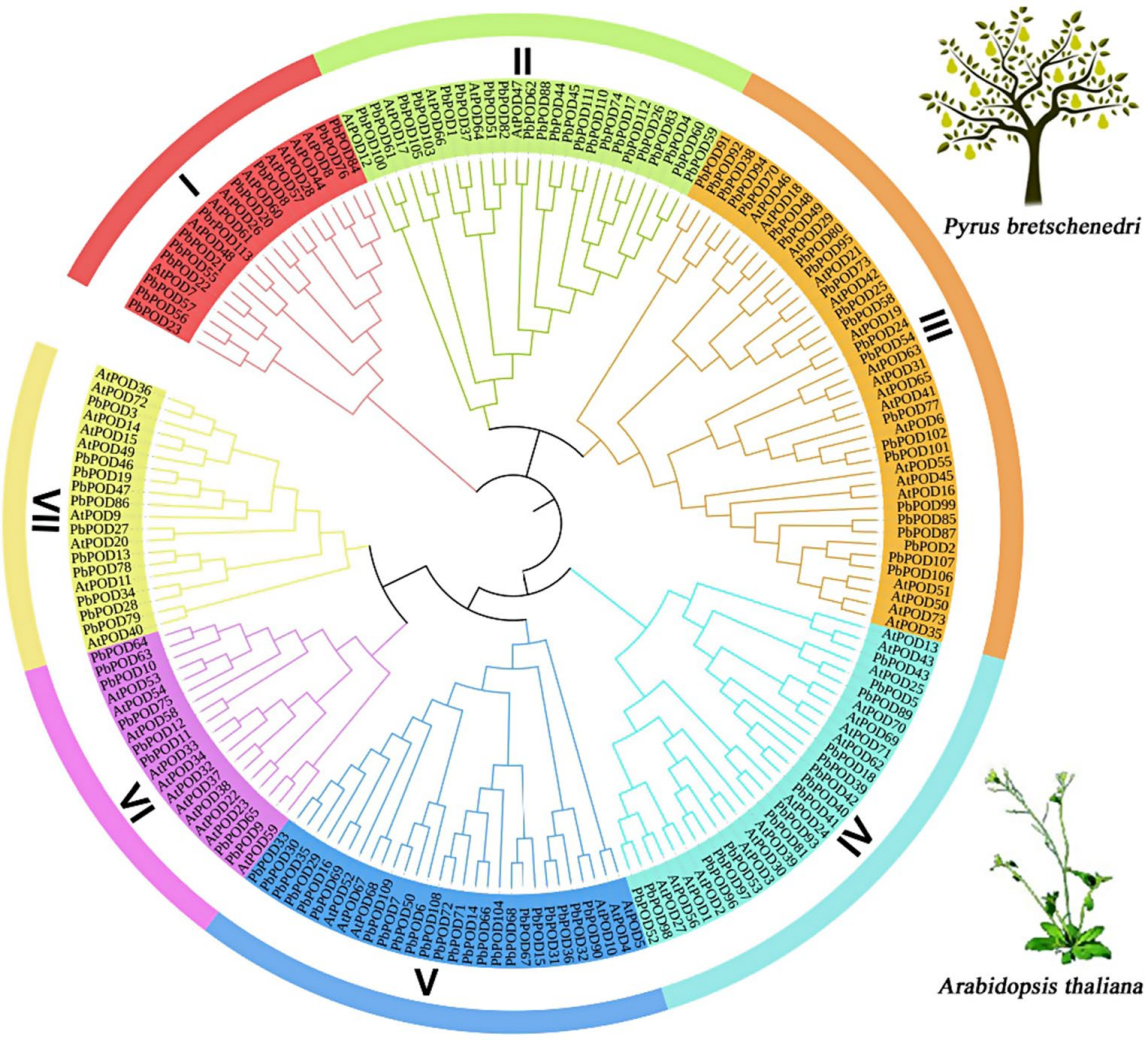
Phylogenetictree o tree of *POD*s from *Pyrus bretschenedri* and *Arabidopsis thaliana*. The analysis utilized full-length protein sequences of the *POD* genes and was conducted using the Molecular Evolutionary Genetics Analysis version 7.0 (MEGA 7.0) software. The phylogenetic reconstruction was performed employing the Maximum Likelihood (ML) statistical method. Branch lines of subtrees are colored, indicating different POD subgroups

To explore the evolutionary relationships among the *POD* family genes, we generated a comprehensive phylogenetic tree of the 113 *PbPODs* and 73 *AtPOD*s of *Arabidopsis thaliana* using the maximum likelihood method in MEGA 7.0. The phylogenetic tree revealed that PODs can be additionally classified into seven distinct subgroups (Fig. 1). The findings demonstrated an asymmetrical distribution of *PbPOD* genes in relation to *AtPOD*s. According to our observations, it has been noted that within the molecular genetics of pear and *Arabidopsis*, subgroup 7 exhibited a greater gene count than did the other subgroups. The phylogenetic tree further revealed the proximate genetic associations with Arabidopsis. In the present study, an evolutionary framework was constructed to elucidate the phylogenetic relationships between the POD proteins of *P. bretschneideri* and *A. thaliana*.

Our analytical outcomes segmented the *POD* genes into five subfamilies, namely, I, II, III, IV, V, VI and VII. For a comprehensive exploration of the phylogenetic affiliations and potential functional divergence inherent to *POD* genes, homologs from both the *P. bretschneideri* and *A. thaliana* genomes were selected, which enabled a multifaceted sequence alignment and a subsequent analysis of the phylogenetic architecture, as illustrated in Fig. 1. Considering the variances in protein structural configurations, in the present study, we divided the POD family into five highly conserved, distinct subfamilies. Each subfamily was confirmed to be robust through rigorous bootstrap validation. Based on phylogenetic assessment, the *POD* genes were taxonomically classified into seven clade-based groups. Notably, group VII was populated by a pronounced number of *PbPOD* constituents. In contrast, subfamily I was more sparsely populated, housing only five gene members. Furthermore, almost all genes related to the POD domain were identified (1 or 2 domains). An ancillary aspect of our research centered on the phylogenetic ties of the *POD* genes of *P. bretschneideri* with those of in *A. thaliana*. Conclusive evidence suggested that the *POD* genes from both of these species demonstrated a closely intertwined evolutionary trajectorie, as shown in Fig. 1.

### Chromosomal localization of *PbPOD* genes

**Fig. 2:**
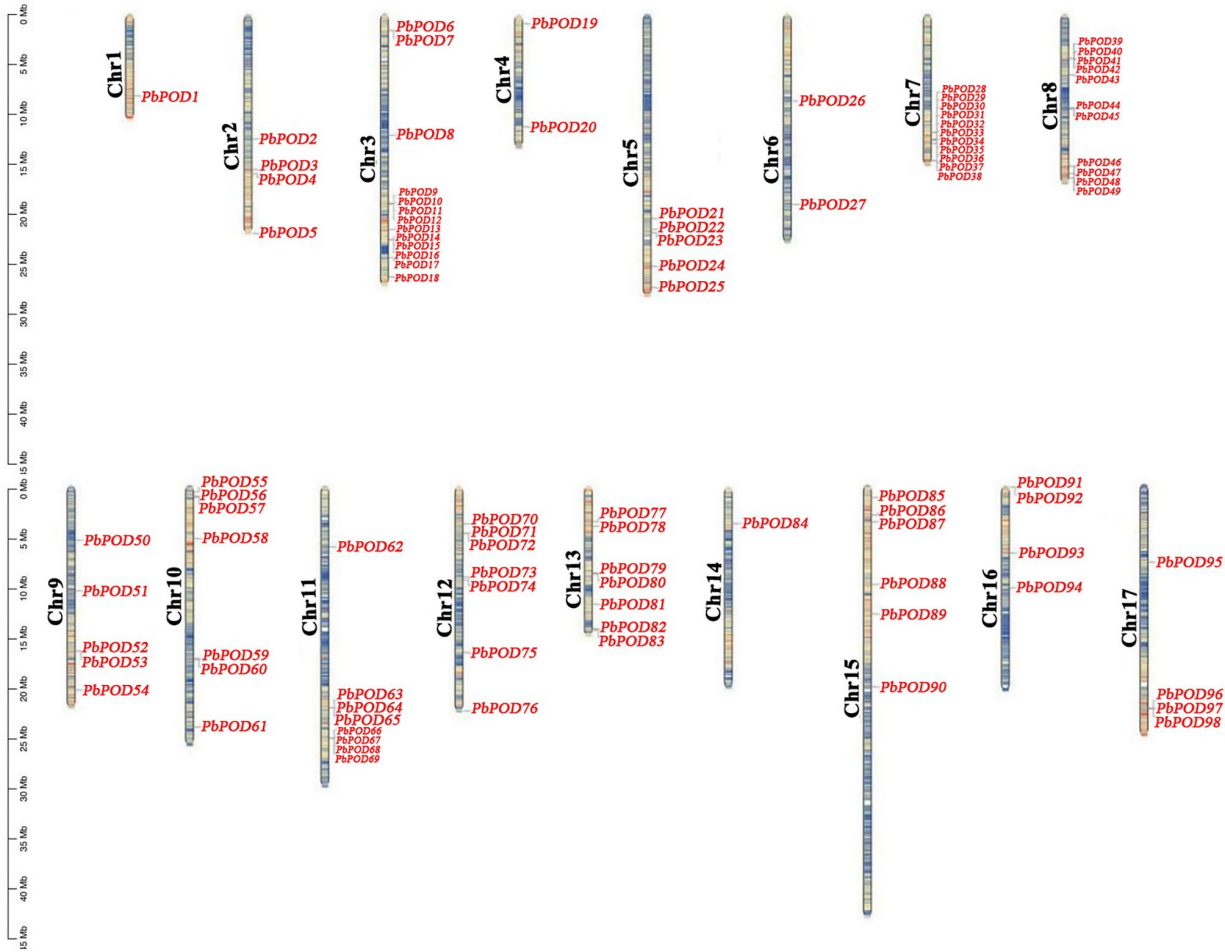
Genomic localization and distribution patterns of *PbPOD* genes in the *P. bretschenedri* genome

Chromosomal mapping of *PbPOD* genes was conducted based on the available genome assembly of *P. bretschenedri*. In total, these genes are distributed across 17 chromosomes, displaying notable disparities in gene density across different chromosomal regions. Specifically, chromosomes 3, 7 and 8 harbor a relatively high density of *PbPOD* genes (Fig. 2). By identifying these uneven distribution patterns and gene clusters, this study sheds light on the underlying genomic architecture that may have implications for the functional specialization and evolutionary history of the *PbPOD* gene family in *P. bretschenedri*. The presence of clustered *PbPOD* genes possibly indicates regions of the genome that have undergone tandem duplication events, which may, in turn, play a role in the rapid diversification and functional expansion of this gene family (Fig. 2).

### Collinearity Ka/Ks analysis of *PbPOD* genes

**Fig. 3:**
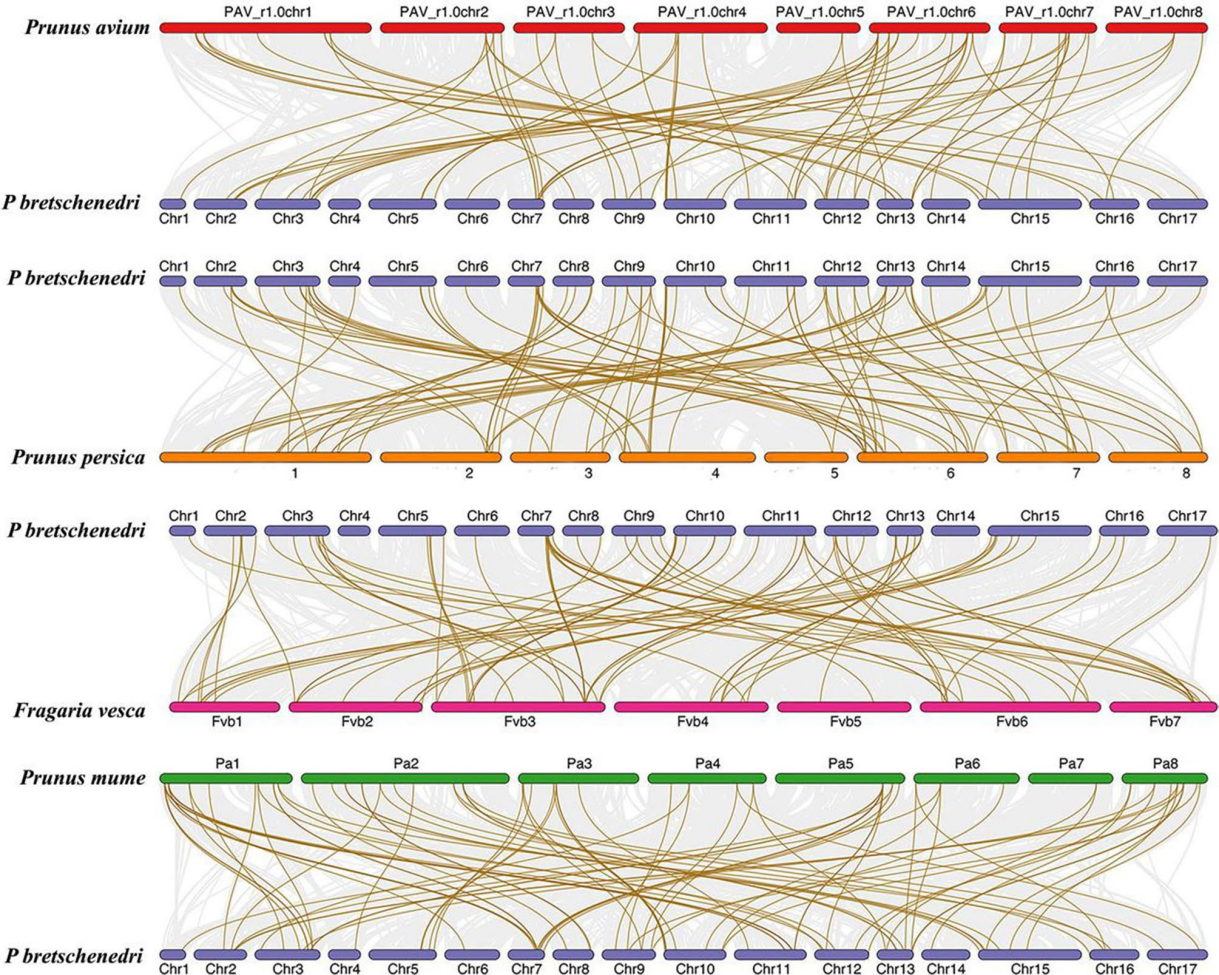
Analysis of syntenic relationships in the *POD* gene family between *P. bretschenedri* and Selected Rosaceae Species: *P. avium*, *P. mume*, *P. persica* and *F. vesca* study explores the syntenic relationships of the *POD* gene family between *P. bretschenedri* (Pb) and four other Rosaceae species—*P. avium* (Pv), *P. mume* (Pm), *P. persica* (Pp) and *F. vesca* (Fv). The analysis employs Bezier lines as a graphical representation to identify and delineate the collinear blocks of genes shared between the two species being compared. These lines serve as the background framework upon which specific gene pairs are mapped

The gene collinearity analysis between *P. bretschenedri*, *P. avium*, *P. persica*, *F. vesca* and *P. mume* was depicted. The findings pertaining to the chromosomal localization of PODs demonstrated heterogeneous distribution patterns, with protein quantities varying from one to seven per chromosome, excluding chromosome 1, which had the lowest number of genes in each species. These distribution patterns were observed across a total of 17 distinct chromosomes, specifically Chr1 to Chr17, within the genome of the pear. Furthermore, the chromosomes exhibited varying gene counts, with Chr7 displaying an impressive number of genes, such as *P. bretschenedri* vs. *P. persica* 13 gene pair clusters, *P. bretschenedri* vs. *P. avium* 9, *P. bretschenedri* vs. *P. mume* 12 pairs and *P. bretschenedri* vs. *F. vesca* has 16 pairs (Fig. 3 and Table S2). In addition, in *P. bretschenedri* and *P. avium*; *P. bretschenedri* and *F. vesca*, exhibited 73 and 86 pairs, respectively. These findings align with the established evolutionary relationships among these species.

**Fig. 4:**
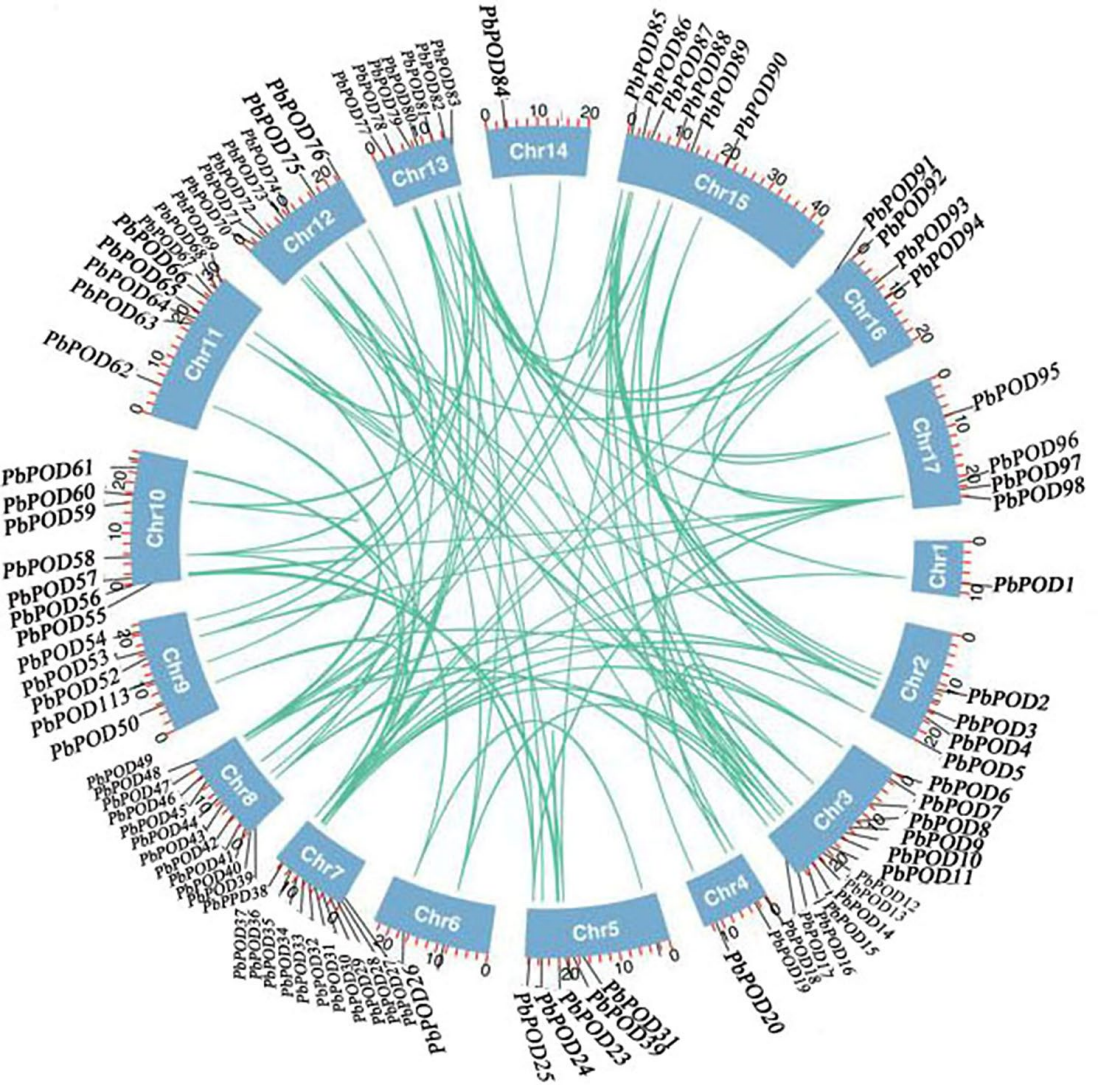
Analysis of gene duplication events, microsyntny of the *POD* gene family in the pear genome. The investigation focused on identifying and characterizing gene duplication events within the *POD* gene family in the pear (*Pyrus bretschenedri*) genome. Duplicated gene pairs within the *POD* gene family are marked by light blue lines, thereby highlighting their genomic locations relative to one another. These light blue lines serve as visual markers that specify duplicated gene pairs, offering a graphical representation of gene duplication events

Hence, within the POD members, notable patterns of genetic variation were detected in the genome of the pear. To gain a deeper understanding of the evolutionary patterns of *PbPOD* genes during the evolutionary process, we investigate more extensive synteny blocks in *P. bretschenedri*. According to collinearity analysis of the *PbPOD* gene, a total of 54 gene pairs were identified to be involved in the replication event (Fig. 4).

Throughout the course of evolutionary events, the genetic elements experience a multitude of selection pressures, encompassing positive selection (with a Ka/Ks ratio greater than 1), purifying selection (with a Ka/Ks ratio less than 1), and neutral selection (with a Ka/Ks ratio equal to 1). The gene duplications of 113 *PbPOD* family members were analyzed. These pairs can be categorized into different types, including 15 pairs that were proximal, 4 pairs that were transposed, 32 pairs of segmental duplications, and 3 pairs that were tandem duplications (Table S3). The experimental findings indicated that the majority of the gene pairs exhibited a Ka/Ks ratio of less than 1.00 (Table S3), implying the presence of purifying selection. This observation further unveiled a restricted level of divergence after duplications of genes. However, it was observed that most of Ka/Ks with values less than 0.6 (Table S3), indicating *PbPOD* gene family may undergo strong negative selection during evolution.

### GO and KEGG and *cis*-regulatory elements analysis in pear

**Fig. 5:**
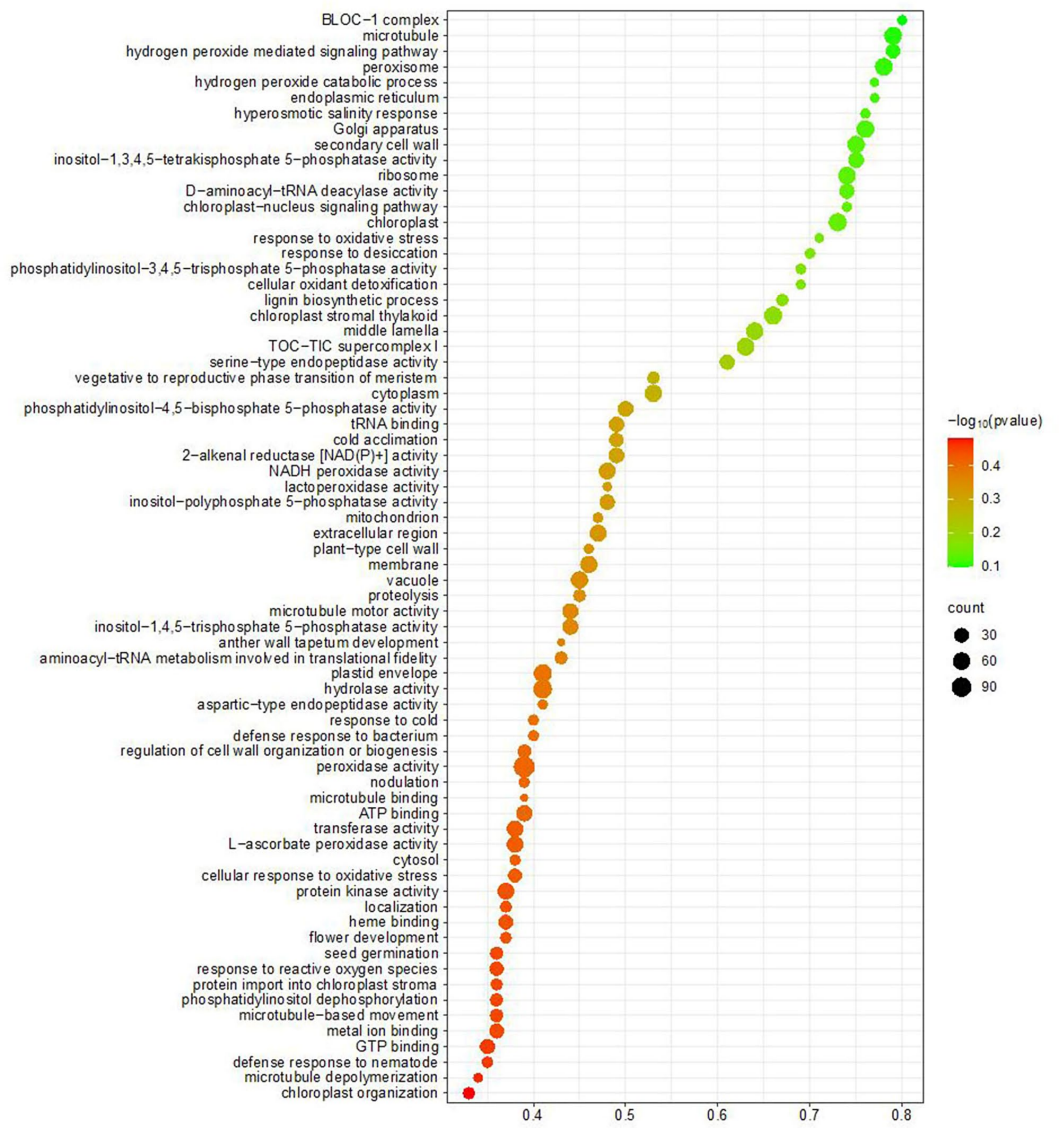
Gene ontology annotation of PbPOD proteins: categorization based on biological process, cellular component and molecular function. The study conducted a comprehensive gene ontology (GO) annotation analysis of PbPOD proteins, aiming to characterize these proteins within the framework of three main GO categories: biological process (BP), cellular component (CC) molecular function (MF). The abscissa of the graphical representation quantifies the proportion of predicted PbPOD proteins that fall under each respective GO term, expressed as a percentage

The GO enrichment analysis was conducted to elucidate the functional regulatory mechanism of POD genes. The observation revealed the presence of three distinct subgroups, namely cellular components, molecular functions, and biological processes (Fig. 5). In the BP processes, the GO terms hydrogen peroxides catabolic process (GO:0042744), response to oxidative stress (GO:0006979); cellular oxidant detoxification (GO:0098869); cellular oxidant detoxification (GO:0098869); hydrogen peroxide catabolic process (GO:0042744); response to oxidative stress (GO:0006979); and cellular oxidant detoxification (GO:0098869) exhibit significant enrichment. In a similar manner, the GO terms associated with CC processes and MF primarily pertain to molecular components such as lactoperoxidase activity (GO:0140825); heme binding (GO:0020037); metal ion binding (GO:0046872); heme binding (GO:0140825); metal ion binding (GO:0046872). In the cellular component, extracellular region (GO:0005576); plant-type cell wall (GO:0009505); membrane (GO:0016020); extracellular region (GO:0005576); vacuole (GO:0005773). The GO terms for molecular function (MF), cellular component (CC), and biological process (BP) indicate the significant involvement of PODs in diverse grapevine activities. Furthermore, the KEGG enrichment analysis revealed the presence of three prominent pathways within the grapevine’s PODs, namely “Biosynthesis of other secondary metabolites,” “Phenylpropanoid biosynthesis,” and “Metabolism” (Table S4).

**Fig. 6:**
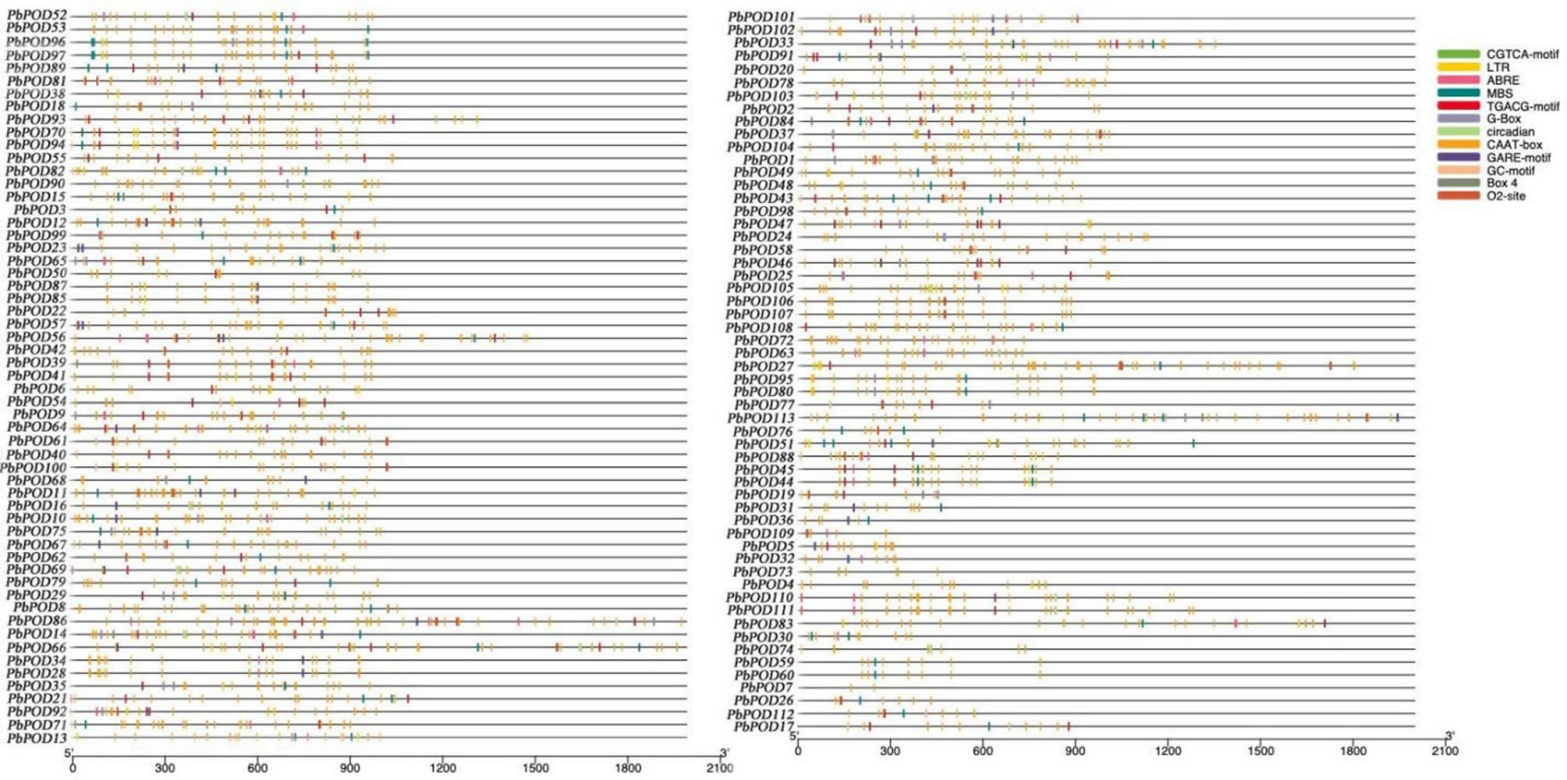
Cis-acting element analysis of *PbPOD* dene promoters: characterization and representation of diverse regulatory elements. Visualization of *cis*-acting elements in *PbPOD* promoters. The study conducted an extensive analysis to characterize *cis*-acting elements within the promoters of *PbPOD* genes. These elements are crucial for understanding the regulation of gene expression. In the graphical representation, different types of *cis*-acting elements are denoted by varying colors, as outlined in the color key provided on the left-hand side of the figure

Furthermore, the *cis*-acting elements located in the promoter region of POD members were analyzed utilizing the PlantCARE database. In a concise manner, the majority of the genes primarily engaged in the regulation of light through significant regulatory components such as, (G-Box, GT1-motif, AE-Box, and GATA-motif), subsequently influenced by hormones (TGACG-motif, CGTCA-motif, GARE-motif, and ABRE), stress and other regulatory factors (o2-site, LTR, CCAAT-Box, ARE, CAT-BOX), and circadian rhythms. CGTCA-motif (146), LTR (59), GARE-motif (36), ABRE (108), MBS (96), Box 4 (15), TGACG-motif (144), G-Box (41), O2-site (28), GC-motif (17), circadian (20), and CAAT-box (1869). The present study provides an examination of the multifaceted functions of POD members and their indirect participation across multiple biotic and abiotic hormone signaling pathways (Fig. 6 and Table S5).

### Analysis of *POD* gene expression in different organs of pear

**Fig. 7:**
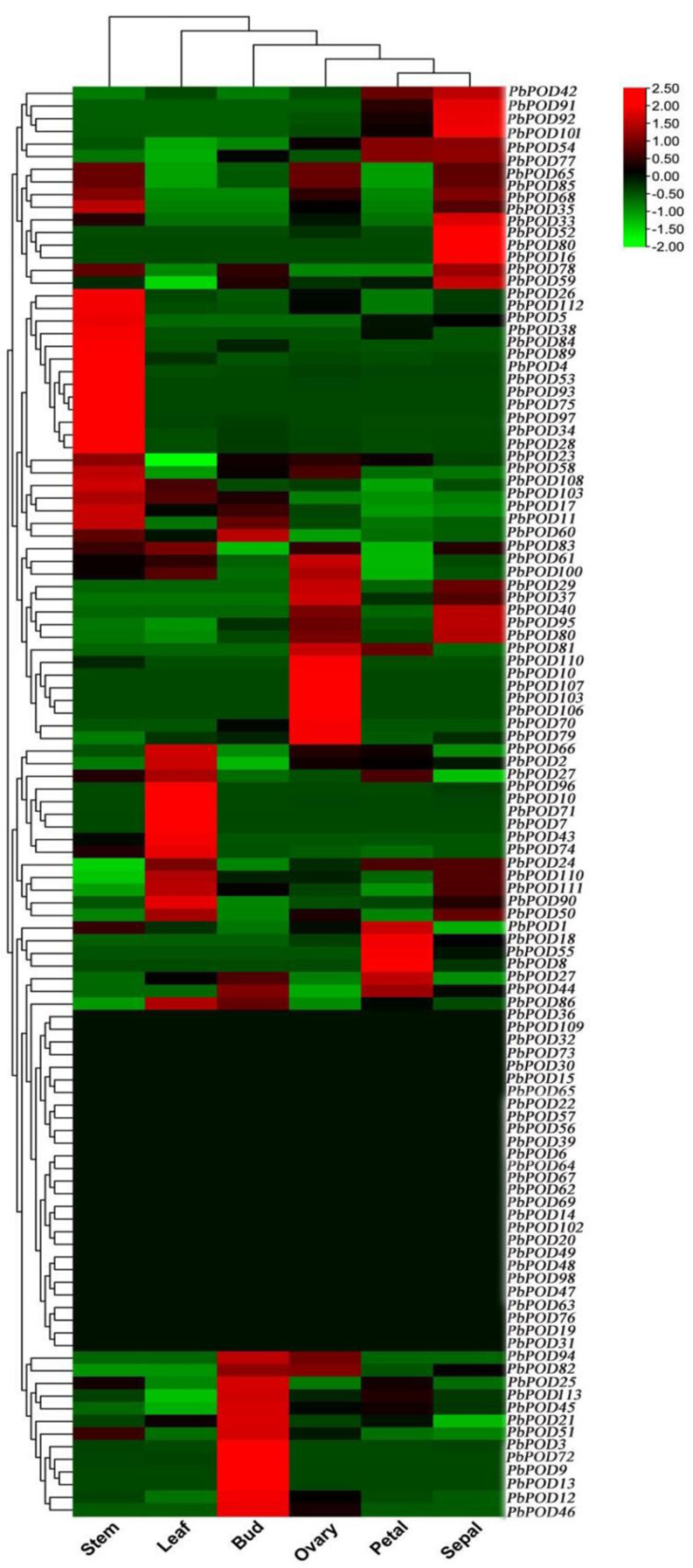
Heatmap clustering analysis in *PbPOD* genes expression in stem, leaf, bud, ovary, petal, and sepal). The scale bar serves as a visual guide for interpreting the degree and direction of gene expression changes. Moreover, the FPKM normalization method ensures that the expression levels are comparable across different genes and treatment durations

In the current study, the investigation focused on the expression profiling of all 113 *PbPOD* genes in pear. These PODs were derived from 6 different organs and tissues (stem, leaf, bud, ovary, petal, and sepal), and their expression patterns were analyzed. The RNA-seq data were obtained from the NCBI database. To depict the spatiotemporal expression pattern, a graphical representation in the form of a heatmap was constructed (Fig. 7). This heatmap was based on the FPKM values, which were logarithmically transformed of the 113 *PbPOD* (*P. bretschenedri* Peroxidase) genes. The specific details of these genes can be found in Table S6. The expression levels of 16 *PbPOD* genes (*PbPOD4*, *PbPOD5*, *PbPOD11*, *PbPOD17*, *PbPOD26*, *PbPOD28*, *PbPOD34*, *PbPOD38*, *PbPOD53*, *PbPOD75*, *PbPOD84*, *PbPOD89*, *PbPOD93*, *PbPOD97*, *PbPOD108*, and *PbPOD112*) exhibited significant and highest expressions in stem, and 10 *PbPOD* (*PbPOD1*, *PbPOD2*, *PbPOD7*, *PbPOD10*, *PbPOD24*, *PbPOD43*, *PbPOD66*, *PbPOD71*, *PbPOD90*, and *PbPOD96*) genes were highly expressed in leaves, while six genes, *PbPOD3*, *PbPOD9*, *PbPOD12*, *PbPOD13*, *PbPOD46*, and *PbPOD72* were highly expressed in buds. In addition, some *PbPOD* genes were significantly expressed in ovary, petal, and sepal, such as *PbPOD10*, *PbPOD55*, and *PbPOD80*. These results indicated that their crucial functions in pear. As well as 27 genes out of 113 no significant expression in any stage. On the other hand, some genes showed lower and no significant expression (*PbPOD41*, *PbPOD52*, *PbPOD64*, *PbPOD66*, *PbPOD78*, and *PbPOD106*) but after hormonal treatment these showed higher expression of different time interval. These results imply their genes have potential involvement in stem, leaf, bud, ovary, petal, and sepal and fruit development stages. Furthermore, the remaining genes exhibited either moderate or weak levels of expression abundance in all the chosen tissues and organs, suggesting their restricted responsiveness in pear plants.

### Expression of *PbPOD* genes during fruit development and under abiotic stress

**Fig. 8:**
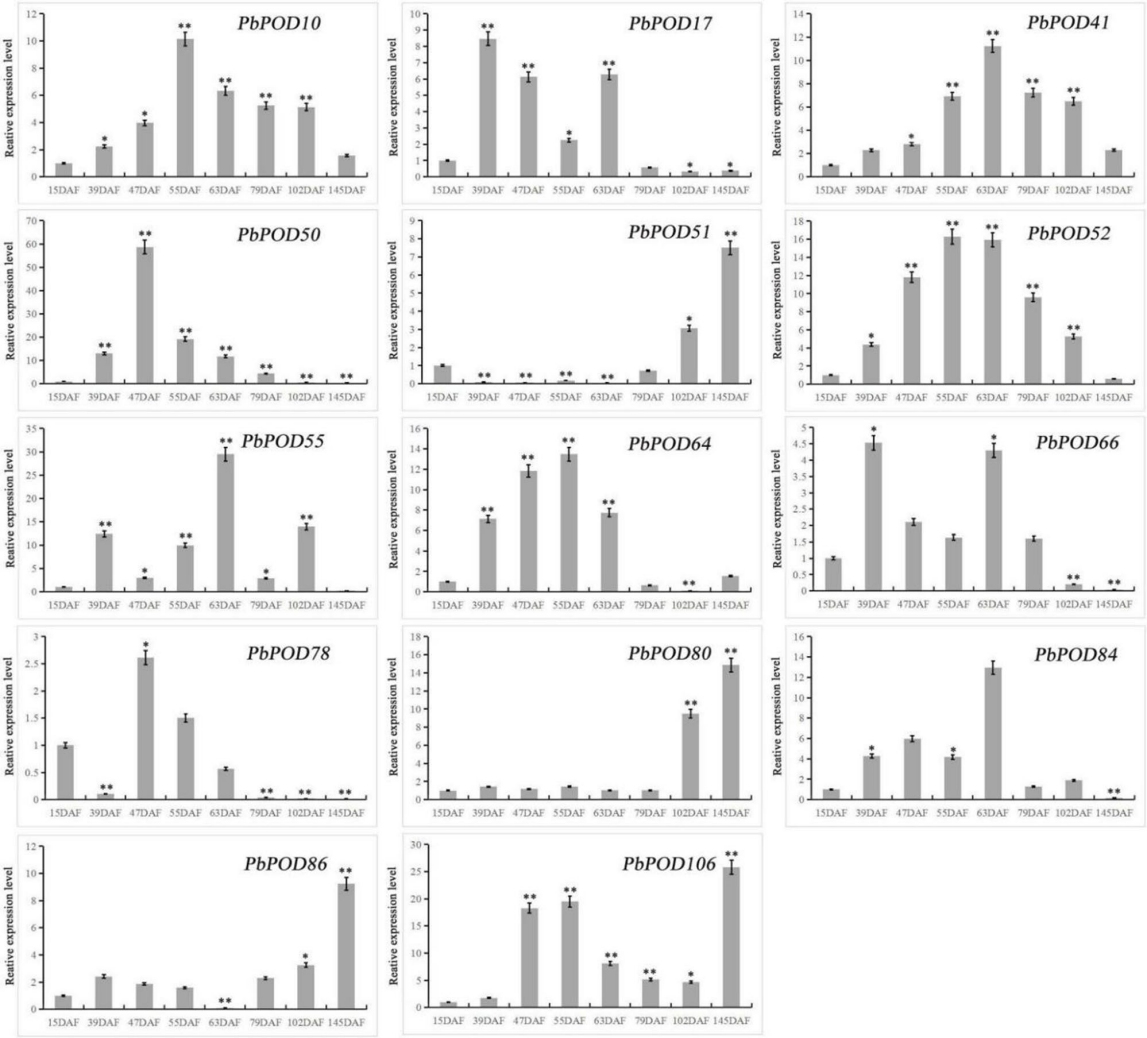
The relative expression of the *PbPOD* gene response during different development stages of fruit (15 DAF, 39 DAF, 55 DAF, 79 DAF, 102 DAF, and 145 DAF) was measured using qRT-PCR. The results were normalized using an internal control, specifically the tubulin gene. The standard error (SE) represented by the error bars is based on three biological replicates. **Signifcant diference (*P* < 0.01), *signifcant diference at *P* < 0.05

To explore the involvement of *PbPOD* genes in different developmental stages involving 15 DAF, 39 DAF, 47 DAF, 55 DAF, 63 DAF, 79 DAF, 102 DAF, and 145 DAF qRT-PCR expression analysis was performed. In the ontogenetic stages of *Pyrus bretschneideri* fruit, the expression patterns of the *PbPOD* genes exhibited heterogeneity (Fig. 8). Specifically, *PbPOD10*, *PbPOD52*, and *PbPOD64* demonstrated an upward transcriptional trajectory, peaking at 55 DAF, followed by a subsequent decrease. Conversely, *PbPOD50* and *PbPOD78* exhibited peaks at 47 DAF, and *PbPOD41* exhibited a peak at 79 DAF, respectively, while *PbPOD51*, *PbPOD80*, *PbPOD85*, and *PbPOD106* exhibited a peak at 145 DAF. Taken together, these results collectively suggest that *POD* gene family members may play a putative role in modulating the development and growth processes of pear fruit.

**Fig. 9:**
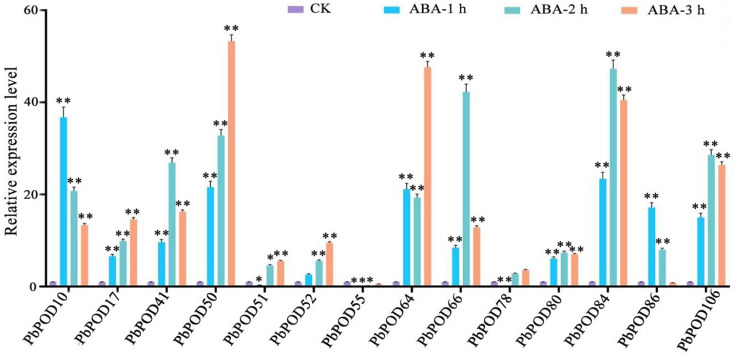
The relative expression of the *PbPOD* gene in response to abscisic acid (ABA) hormonal stress was measured using quantitative PCR (qPCR). The results were normalized using an internal control, specifically the tubulin gene. The standard error (SE) represented by the error bars is based on three biological replicates. **Signifcant diference (*P* < 0.01), *signifcant diference at *P* < 0.05

**Fig. 10:**
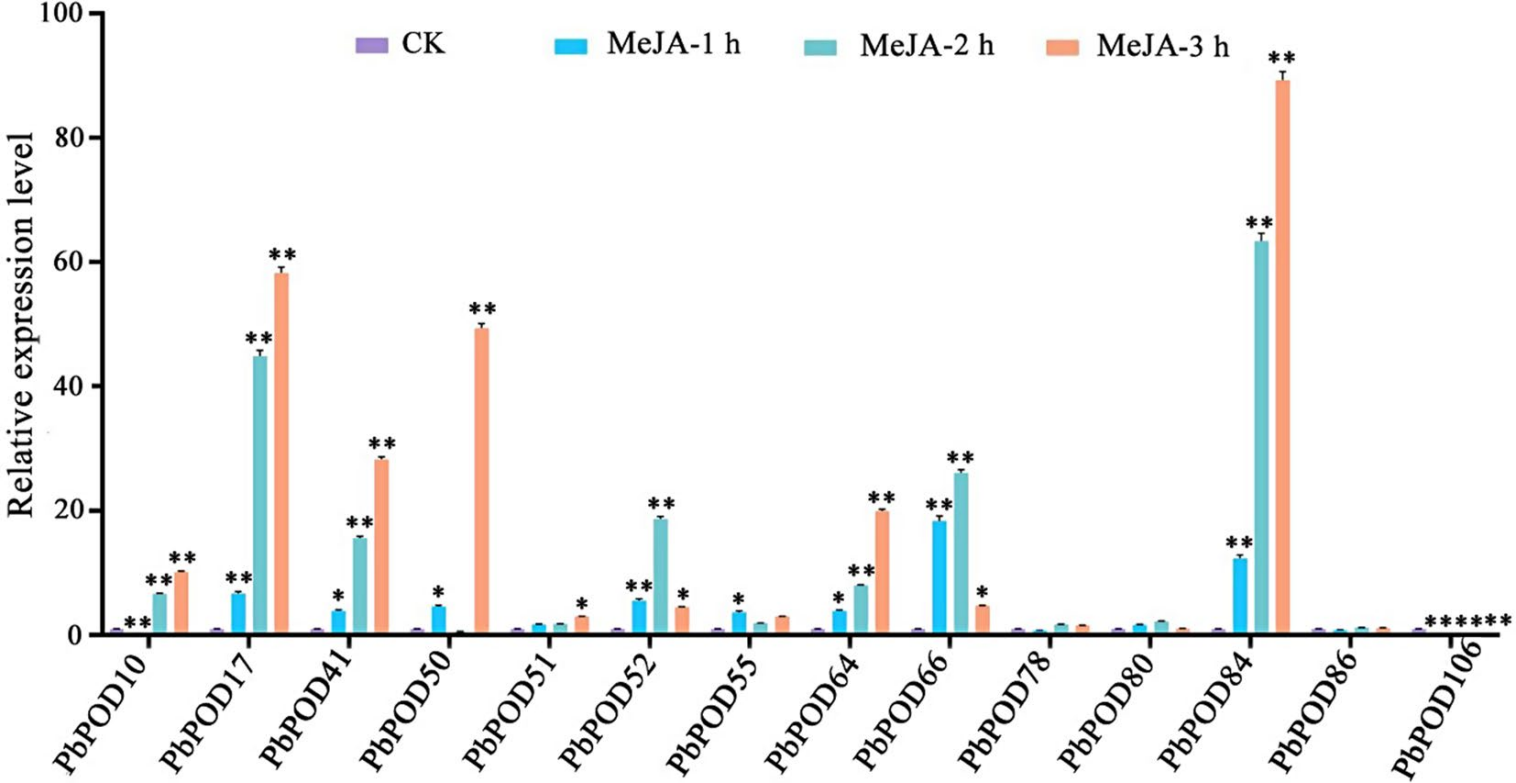
The relative expression of the *PbPOD* gene in response to methyl jasmonate (MeJA) hormonal stress was measured using quantitative PCR (qPCR). The results were normalized using an internal control, specifically the tubulin gene. The standard error (SE) represented by the error bars is based on three biological replicates. **Signifcant diference (*P* < 0.01), *signifcant diference at *P* < 0.05

**Fig. 11:**
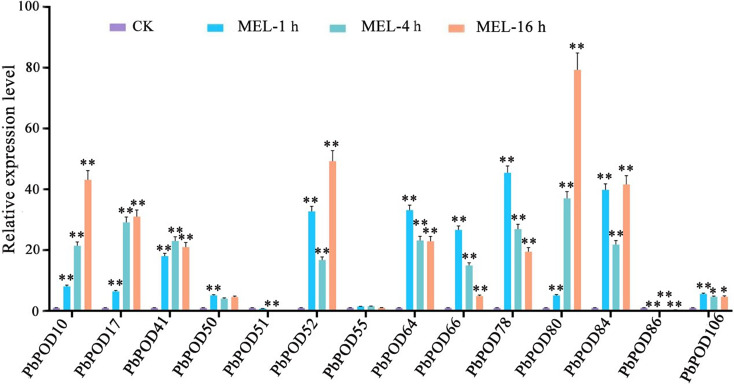
The relative expression of the *PbPOD* gene in response to melatonin (MEL) hormonal stress was measured using quantitative PCR (qPCR). The results were normalized using an internal control, specifically the tubulin gene. The standard error (SE) represented by the error bars is based on three biological replicates. **Signifcant diference (*P* < 0.01), *signifcant diference at *P* < 0.05

**Fig. 12:**
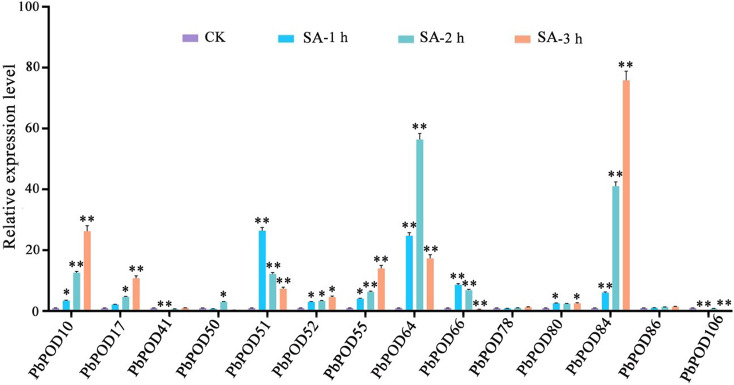
The relative expression of the *PbPOD* gene in response to salicylic acid (SA) hormonal stress was measured using quantitative PCR (qPCR). The results were normalized using an internal control, specifically the tubulin gene. The standard error (SE) represented by the error bars is based on three biological replicates. **Signifcant diference (*P* < 0.01), *signifcant diference at *P* < 0.05

We also explored the involvement of *PbPOD* genes in various abiotic stresses, including exposure to melatonin (MEL), salicylic acid (SA), abscisic acid (ABA), and methyl jasmonate (MeJA) via qRT-PCR analysis and selected 14 genes based on phylogenetic analysis. These genes were exposed to MEL, SA, MeJA and ABA stress treatments. The findings revealed that all these genes exhibited diverse responses, manifesting either low, high, or moderate expression levels in comparison to the control conditions. Under conditions of hormonal stress, it was observed that all genes exhibited increased expression levels. Subsequent to the exogenous hormonal treatments of SA, MeJA, and ABA at intervals of 1 h, 2 h and 3 h, and of MEL at 1 h, 4 h, and 16 h a marked variability in the transcriptional profiles of *POD* genes was observed, as shown in Figs. 9, 10, 11 and 12.

In *Pyrus bretschneideri* fruit subjected to ABA treatment, a substantial upregulation in the expression of *PbPOD10* and *PbPOD86* were evident just one-hour post-application, exhibiting fold-increases of 36.78 and 18.62, respectively, in comparison to the control at 0 h. Additional results indicated that the expression of *PbPOD41*, *PbPOD66*, *PbPOD84*, and *PbPOD106* reached their maximum at 2 h, *PbPOD17*, *PbPOD50*, and *PbPOD64* reached their highest at 3 h, with fold-increases of 14.01, 56.38, and 52.01, respectively (Fig. 9).

In the cohort treated with MeJA, a pattern analogous to that observed with ABA was discerned. Specifically, *PbPOD55* was notably upregulated within an hour of treatment, displaying fold-changes of 3.71. Moreover, the expression levels of *PbPOD52* and *PbPOD66* peaked at the 2 h interval, with fold-changes of 18.7 and 26.13, respectively, in contrast to control. Finally, *PbPOD10*, *PbPOD17*, *PbPOD41*, *PbPOD50*, *PbPOD51*, *PbPOD64*, and *PbPOD84* demonstrated a peak in expression at 3 h, exhibiting fold-changes of 10.1, 58.29, 28.28, 49.35, 3.01, 19.97 and 89.30, respectively, as outlined in Fig. 10.

In MEL treatment group, the expression level of *PbPOD50*, *PbPOD64*, *PbPOD66, PbPOD78*, and *PbPOD106* reacheed their maximum at 1 h, while the expression levels of *PbPOD41* reached its highest at 2 h. Simultaneously, *PbPOD10*, *PbPOD17*, *PbPOD52*, *PbPOD80*, and *PbPOD84* manifested peak expression at the 3 h mark, with fold-changes of 43.12, 31.02, 49.28, 79.31, and 41.59, respectively, relative to the control levels. Conversely, the expression of *PbPOD86* was conspicuously inhibited among 1, 2, and 3 h post-treatment as compared to ABA and MeJA as shown in Fig. 11.

In the SA treatment group, expression profiles revealed that *PbPOD51* experienced significant upregulation at 1 h post-application, and reached the highest level with fold-increases of 26.38 higher than the control. At the 2 h mark, the transcriptional activity of *PbPOD64* reached its apex, with fold-changes of 56.58. Furthermore, at 3 h post-treatment, *PbPOD10*, *PbPOD17*, *PbPOD52*, *PbPOD55*, and *PbPOD84* displayed peak expression levels, with respective fold-increases of 26.23, 10.82, 4.59, 14.01 and 75.85, as demonstrated in Fig. 12. Besides, compared with the control group, there was no significant change in the expression levels of *PbPOD78* and *PbPOD86* during the entire treatment process.

Furthermore, an analysis of the correlation based on relative expression revealed a predominantly strong positive correlation, although some genes exhibited an inverse correlation. In summary, these results highlight the differential expression patterns of *POD* genes in response to multiple stressors, underscoring their potential significance in promoting plant growth and resilience.

## Discussion

Due to the substantial role of Class III PODs in various physiological processes, including their involvement in responding to both biotic and abiotic stresses [1, 13]. Therefore, this is very necessary to systematic exploration of the potential functions of *POD* genes in pear, a crucial crop. In this research study, we successfully identified 113 *PbPOD* genes within the pear genome. This observation indicated that pear possesses more POD members compared to some reported species, such as *Arabidopsis* (73) [14] and *Daucus carota* (102) [1]. However, a smaller count compared to other species, such as rice (138) [13], tobacco (310) [15], and wheat (374) [16]. Thus, this indicates a significant expansion of the *POD* gene family in pear, tobacco, and wheat compared to other plant species.

During plant evolution, gene duplications have signifcant impact on the expansion of gene families [17]. In this study, we found that *PbPOD* genes are usually distributed in clusters on chromosomes, forming multiple gene clusters (Fig. 2), which is similar to the distribution of *PpPOD* genes on peach chromosomes [18]. For example, chromosomes 3 and 7 contained 13 and 11 *PbPOD* genes, respectively (Fig. 2), which are adjacent and closely arranged, and have a very close evolutionary relationship with each other. In addition, we identified 54 duplication gene pairs from a total of 113 *PbPOD*s (Fig. 3, Table S3). We believe that segmental duplication was considered the main driving force for the expansion and evolution of the *PbPOD* gene family. For example, 63% (34) of duplicated gene pairs were observed to be caused by segmental duplication, and 3 pairs (6%) of duplication pairs evolved through tandem duplication events. Our research findings are similar to the previous reports that segmental duplication is the main driving force for the evolution and expansion of the *POD* gene family in soybean [1] and rice [13].

To further analyze whether these tandem or segmental duplication genes are subjected to selection pressure during the evolutionary process, we calculated the Ka and Ks values of these genes. Our results indicate that 95% (35) of duplication genes have a Ka/Ks < 1, and only 2 pairs of duplicate genes have the characteristic of Ka/Ks > 1(Table S4). Similar results have also been found in studies on the evolution of soybean [1], tobacco [15], and *Passiflora edulis* [19]. These results indicated that the *PbPOD* gene family mainly evolved through positive selection, and positive selection accelerated the evolution of the *POD* gene family in pears.

As Biłas et al. [20] described that the regulation of gene expression often necessitates the synergy of multiple *cis*-acting elements. Therefore, the identification of *cis*-elements in *PbPOD* provided a good opportunity for further understanding the possible transcriptional regulation of these genes in various physiological processes in the future. In our study, a variety of frequently occurring *cis*-acting elements, including MBS, ARE, and ABRE, were investigated in the promoter regions of *PbPOD* (Fig. 6). In addition, we also found that almost all *PbPOD* genes contain at least one promoter *cis*-acting element associated with stress and plant hormones. These results indicated that the *PbPOD* gene family might be under the regulatory influence of specific plant hormones, potentially playing a role in hormone-driven growth, development, or stress response mechanisms. Similar to our findings, Xiao et al. [21] previously found similar types of *cis*-acting elements in the *POD* gene promoter region of grape species in the Rosaceae family. Besides, many previous studies have shown that *POD* regulates multiple target genes [22], and the loss of their function affects many physiological processes and responses to different plant stresses, leading to phenotypic changes [23, 24]. In addition, recent studies have also elucidated the role of plant peroxidases in various intracellular mechanisms during plant development and maturation, as well as their response to abiotic and biological environmental pressures [25, 26].

RNA-seq data is generally used to study the mRNA expression levels transcribed by specific plant tissues or cells over a certain period of time, and then analyze relevant genes and phenotypes [27]. In our research, we used the obtained RNA-seq data from different pear tissues to investigate the possible functions of *PbPOD*s (Fig. 7). Our results found that *PbPOD*s exhibit tissue-specific expression, indicating that *PbPOD*s have different functions. The RNA-seq results showed that among these 113 *PbPOD*s, some genes were not expressed or had low expression levels in the pear tissue. We speculate that these *POD* genes may play a small role in pear growth and development. In addition, *PbPOD26*, *PbPOD38*, *PbPOD84*, and *PbPOD112* were highly expressed in stems of tissues with higher lignin content. The results indicated that those genes may play important roles in pear xylem synthesis. Besides, *PbPOD27*, *PbPOD44*, and *PbPOD113* were most expressed in pear bud and petal, indicating that they may be related to pear bud extension and flowering formation. Furthermore, some genes were expressed in various tissues, such as *PbPOD65*, *PbPOD83*, *PbPOD85*, and *PbPOD87*, indicating that these *POD* genes may have a significant impact on the growth and development process of pears. In summary, our results indicate that the *POD* family genes play an important regulatory role in the growth and development of pears.

Drought, low temperature, high salinity and other abiotic stresses are serious natural disasters for plants, which seriously affect their growth and development [28]. Previous reports on stress treatment have shown that under drought, low temperature, and other stress conditions, the expression of plant *POD* genes undergoed significant changes [1, 19, 30, 30]. However, there is limited research on the response of *POD* genes to hormones in plants. Previous researches have found that genes associated with hormone stress responses are typically implicated in orchestrating plant stress responses [31, 32]. These responses are orchestrated through intricate hormone signaling pathways. For instance, studies on *PbPOD* genes in *Pyrus bretschneideri* unveiled a plethora of MeJA and ABA-responsive *cis*-acting elements (Table S5). Moreover, our research unveiled several common *cis*-acting elements in the POD promoter region, suggesting potential hormone-induced modulation of these genes. In order to illuminate the differential expression patterns of the pear *POD* genes, we conducted qRT PCR experiments under different hormone treatments (MeJA, ABA, and SA) (Figs. 9, 10, 11 and 12). Among the *PbPOD* genes, a predominant number of manifested conserved domains, highlighting their potential hormone responsiveness. Interestingly, the sensitivity of *PbPOD* genes to hormones varied considerably, these treatments strongly upregulated some *PbPOD* genes, indicating that members of the POD family may have roles in abiotic stress response mechanisms. These results provide evidence that *POD* members can participate in responses to abiotic stress, particularly hormone stress. We speculated that these three hormones may directly or indirectly regulate the transcription level of pear *POD* genes. In the future, further studies are imperative to elucidate the precise regulatory influence of these hormones on *PbPOD* transcription levels. In addition, we also hope to explore the effect of exogenous hormones on the regulation of pear *POD* expression levels, in order to determine whether the growth and development of pear fruits can be altered by regulating exogenous hormones.

In brief, there is a certain correlation between hormone response and plant resistance to abiotic stress. For example, gene expression patterns related to ethylene suggest that ethylene may indirectly participate in the induction of dormancy genes, thereby enhancing the cold resistance of *P. mume* [33]. Moreover, researchers have also observed this phenomenon in the expression pattern of the *TALE* gene, where the expression of TALE is not only regulated by certain hormones, but sometimes also influenced by some abiotic stresses [34, 35]. Aleem’s research found that overexpression lines of *GsPOD40* exhibit significantly higher drought tolerance compared to wild-type (WT) plants under stress treatment [1]. These findings suggest that different *POD* genes have different functions in various biological processes, including biotic and abiotic stress responses as well as hormone signaling pathways.

## Conclusions

This study focused on the Class III peroxidase (POD) family in Chinese pear (*Pyrus bretschenedri*), an area with limited prior research. The researchers characterized 113 *PbPOD* genes and categorized them into distinct subfamilies, revealing the role of segmental duplication events in their expansion. GO, KEGG enrichment along *cis*-acting elements was also performed in pear. The study also examined functional diversity and expression patterns, highlighting the multiple gene responsiveness to stress and their importance in fruit development. The findings position *PbPOD* genes as promising subjects for further research and potential tools for enhancing fruit quality through molecular breeding. Overall, the study advances our understanding of *POD*s gene roles in plant development, hormone signaling, and stress responses in the context of Chinese pear.

## Material and method

### Identification of *POD* gene family and analysis physical properties in *P. bretschneideri*

With the use of the BioEdit tools, we utilized seventy-three (73) sequences of *Arabidopsis* against the pear genome to identify the *POD* genes with an E-value of 1e^− 5^. Moreover, the sequences of pear and *Arabidopsis* were retrieved from online sources, such as the Pear Genome (http://peargenome.njau.edu.cn) [36] and TAIR genome databases (http://www.arabidopsis.org) [37], respectively. The SMART database (http://smart.embl-heidelberg.de/) [38] and NCBI-Conserved Domain database (https://www.ncbi.nlm.nih.gov/Structure/cdd/wrpsb.cgi) were used for the verification of domain composition [3]. Sequences with obvious errors in length as well as sequences without POD domains were eliminated before carrying out the analysis. Several physicochemical analyses viz., isoelectronic points (PIs), molecular weight (MW), and GRAVY, were performed for each gene of *POD* gene by ExPASY PROTPARAM tools (https://web.expasy.org/protparam/) [39].

### Sequence alignment and phylogenetic analysis

All POD full-length amino acid sequences of pear and *Arabidopsis thaliana* were aligned, as well as downloaded from the *Arabidopsis* (https://www.arabidopsis.org) [7] and pear genome database (http://peargenome.njau.edu.cn/) [40]. The MUSCLE was performed using the MEGA 7.0 version for multiple sequences alignment of PODs for phylogenetic analysis. Using the maximum likelihood method (MLM) and phylogenetic tree was constructed, as well the amino acid substitution model was chosen (Jones, Thorton, and Taylor) [41, 42]. The bootstrap values of one thousand (1000) were used to ensure the reliability of the phylogenetic tree while other parameters were kept as default [43]. Finally, the phylogeny tree was constructed through the online itols website (http://itol.embl.de) [44].

### GO and KEGG and subcellular localization of *PbPOD* gene family

The study employed two different online tools, namely the Panther server and the KEGG genome server, to conduct enrichment analyses for Gene Ontology (GO) and KEGG pathways (https://www.genome.jp/kegg/pathway.html) [45–47]. Subsequently, the pathways that showed enrichment were further examined using TBtools software [48]. we further predicted the subcellular localization with the use of the WOLF PSORT (https://wolfpsort.hgc.jp/) online server [49].

### Cis-elements predictions of *PbPOD* gene family

To initiate the analysis, the promoter sequences of *POD* genes, each spanning 2000 base pairs, were first imported into the CDS sequence from the pear genome. Subsequently, various *cis*-regulatory elements were identified within each of these promoter sequences using the PlantPan database (http://plantpan.itps.ncku.edu.tw/plantpan4/index.html) [50].

### Gene collinearity analysis and chromosomal mapping of *PbPOD* gene

In this study, the researchers accessed the pear genomic database to determine the chromosomal positions of *POD* genes and visualized them by TBtool. They then employed this available information to create chromosomal maps for these genes. Furthermore, to analyze the gene collinearity relationship between *Pyrus bretschenedri*, *Prunus avium*, *Prunus mume*, *Prunus*, and *persica*, used the Collinearity Scan Toolkit (https://github.com/wyp1125/MCScanX) [51].

### Duplication events and calculation of non-synonymous (Ka) and synonymous (Ks)

With the use of the MEGA software (7.0 version), the rate of Ka/Ks was carried out for numbers of the duplicate pairs viz., tandem, dispersed, segmental, and proximal. The method used to find out the ratio of Ks and Ka, followed the Nei-Gojopori method with the bootstrap values of one thousand in MEGA 7.0. The MCScan algorithm (https://github.com/wyp1125/MCScanX) was used to detect the duplication of various types (transposed duplication, dispersed duplication, segmental duplication, and tandem duplication) of *POD* gene pairs.

### Plant material and method

Chinese white pear fruit samples were carefully harvested 39 days after flower (DAF) from Anhui Agricultural University experimental base. To apply specific treatments, melatonin (MEL), salicylic acid (SA), abscisic acid (ABA), and methyl jasmonate (MeJA), a well-documented method described [52] was employed. The treatments salicylic acid (SA), abscisic acid (ABA), and methyl jasmonate (MeJA) were administered at 3 different time points, namely 0 h (control), 1 h, 2 h, and 3 h. As well as melatonin at 0 h (control), 1 h, 4 h, and 16 h. Subsequently, each of the fruit samples was promptly frozen using liquid nitrogen and stored at a temperature of -80 °C to facilitate subsequent in vitro testing.

### Transcriptomic data analysis

To do expression profiling, we obtained RNA sequencing data from the NCBI GEO (https://www.ncbi.nlm.nih.gov/geo/) website on stem, sepal, petal, ovary, bud, and leaves were retrieved through the accession numbers SRR8119906, SRR8119889, SRR8119903, SRR8119895, SRR8119898, and SRR8119907. The quantification of expression levels was performed using FPKM (fragments per kilobase of transcript per million fragments mapped). Finally, heat maps were visualized by using the R package.

### Isolation of RNA and profiling of the *POD* gene family in *P. bretschneideri*

Primer sets meticulously tailored to target specific genes were meticulously designed, and their precision was rigorously assessed using the NCBI Primer Blast tool and Primer Premier 5.0 [53]. The comprehensive list of all these primers is shown in Table S1. For consistency and reliability in this study, the pear tubulin gene (AB239680.1) was judiciously chosen to serve as the reference standard [54]. Subsequently, cDNA synthesis was accomplished with precision, employing approximately 2 mg of total RNA, utilizing the TransScript® One-Step gDNA Removal and cDNA Synthesis SuperMix, sourced from TRANSGEN in Beijing, China. The quantification of gene expression was executed through qRT-PCR, utilizing the LightCycler 480 SYBRGREEN I Master from Roche, USA, in strict accordance with the protocols outlined in [55], and the manufacturer’s instructions. Each individual sample underwent a total of three distinct biological replicates to ensure robust and reliable results. To enable a meaningful comparison with untreated control plants, the gene’s relative expression level was meticulously calculated using the 2^−∆∆^^CT^ method [56].

### Electronic supplementary material

Below is the link to the electronic supplementary material.

Supplementary Material 1

## Data Availability

The datasets analyzed in this article are available in the GenBank of NCBI, and the RNA sequencing datas were obtianed from Gene Expression Omnibus (GEO) of China National GenBank (CNGBdb) with accession number the accession numbers SRR8119906, SRR8119889, SRR8119903, SRR8119895, SRR8119898, and SRR8119907. The other Other amino acid sequences analyzed in this study are listed in the supplement Table 7.
